# An automated patient‐specific segment reduction‐based beam angle optimization technique for deep learning auto‐planning for early breast cancer

**DOI:** 10.1002/acm2.70293

**Published:** 2025-10-14

**Authors:** Michele Zeverino, Gian Guyer, Wendy Jeanneret‐Sozzi, Fernanda Herrera, Francois Bochud, Raphaël Moeckli

**Affiliations:** ^1^ Institute of Radiation Physics Lausanne University Hospital and University of Lausanne Lausanne Switzerland; ^2^ Radiation Oncology Department Lausanne University Hospital and University of Lausanne Lausanne Switzerland

**Keywords:** beam angle optimization, breast cancer radiotherapy, deep learning auto‐planning, dose mimicking

## Abstract

**Background:**

Deep learning (DL)‐based auto‐planning has emerged as a powerful tool for optimizing radiotherapy treatment plans, reducing variability, and improving efficiency. However, current approaches often rely on predefined beam angles and arc spans, which may not be optimal for individual patients. Automated beam angle optimization can further enhance plan quality, particularly in early‐stage breast cancer radiotherapy, where precise beam configurations are crucial for balancing target coverage and organ‐at‐risk (OAR) sparing.

**Purpose:**

This study presents an automated segment reduction‐based beam angle optimization technique to improve DL‐based auto‐planning for radiotherapy in early‐stage breast cancer. The method optimizes arc spans for volumetric‐modulated‐arc‐therapy (VMAT) and beam configurations for intensity‐modulated‐radiation‐therapy (IMRT) to improve dose distribution while reducing OAR exposure.

**Methods:**

Plans using three different irradiation strategies—partial arc VMAT (PA‐VMAT), complex IMRT (C‐IMRT), and simple IMRT (S‐IMRT)—were generated using two full arcs for dose mimicking of the predicted dose, followed by the segment reduction performed using a stepwise PAMU (Product of segment Area and Monitor Units) thresholding approach to determine optimal arc spans and beam angles. These strategies were compared against the standard continuous partial arc VMAT (CPA‐VMAT) technique currently used in our clinical practice. Twenty left‐sided breast cancer patients treated under deep inspiration breath‐hold (DIBH) conditions were included for evaluation. Plan quality was assessed using dosimetric criteria, conformity indices, dose mimicking index (DMI), and statistical comparisons.

**Results:**

PA‐VMAT exhibited superior OAR sparing and the best overall dose mimicking performance, reducing the heart, left lung, and right lung mean doses by 27%, 11%, and 50%, respectively, compared to CPA‐VMAT. C‐IMRT provided the best target coverage but required higher monitor units, while S‐IMRT showed suboptimal dose homogeneity. The automated segment reduction method significantly improved plan efficiency, optimizing beam angles without requiring manual intervention.

**Conclusion:**

This study demonstrates the feasibility of an automated segment reduction‐based optimization technique for DL auto‐planning in early‐stage breast cancer. PA‐VMAT emerged as the preferred strategy, balancing plan quality, delivery efficiency, and OAR sparing. The proposed approach enhances treatment planning flexibility and will be incorporated into future clinical practice.

## 1 INTRODUCTION

Radiation therapy following breast‐conserving surgery, combined with chemotherapy, hormonotherapy, and/or immunotherapy, remains the standard of care for breast cancer patients, significantly reducing the risk of recurrence.^1^, ^2^, ^3^ Depending on tumor staging, several radiation techniques are available. These range from traditional forward‐planning 3D conformal tangential fields (with or without field‐in‐field fluence‐based optimization)^4^, ^5^ for early‐stage breast cancer to more advanced inverse planning techniques such as intensity‐modulated radiation therapy (IMRT), volumetric modulated arc therapy (VMAT), or a combination of both,^6^, ^7^, ^8^, ^9^, ^10^ which are also applicable to advanced diseases, including cases with nodal involvement. Numerous dosimetric studies have evaluated the advantages and limitations of each method. In summary, tangential techniques are effective for sparing organs at risk (OARs) but may result in dose inhomogeneity within the planning target volume (PTV). Conversely, inverse planning techniques offer superior dose homogeneity and conformity for the PTV, albeit at the cost of increased low‐dose exposure to surrounding OARs.^4^, ^5^

Recently, various machine learning (ML) auto‐planning techniques, including knowledge‐based^11^, ^12^ and deep‐learning (DL) methods,^13^, ^14^, ^15^ have been clinically implemented. These approaches have demonstrated their ability to reduce inter‐planner variability while maintaining or even improving the quality of dose distributions compared to manual plans across all irradiation techniques employed in breast cancer treatment.

Regardless of whether the planning approach is manual or automated, the planner must typically configure the number of beams or arcs, as well as their angles or arc spans. This is generally achieved either by manually selecting angles to minimize OARs irradiation (e.g., contra‐lateral breast and ipsilateral lung for breast planning) or by using deterministic algorithms that automatically identify optimal beam configurations to enhance OARs sparing,^16^, ^17^, ^18^, ^19^ although some authors have also explored the use of machine learning techniques.^20^, ^21^

Among automated approaches, commercially available DL solutions rely on an automated pipeline comprising three sequential steps to generate clinical plans: dose prediction, post‐processing of the predicted dose, and dose mimicking of the post‐processed dose.^15^, ^22^ The predicted dose (PD) is the output of a pre‐trained 3D U‐NET autoencoder model, which is fed with the patient's CT scan and a specific set of regions of interest (ROI) defined for the model. The PD may undergo an optional post‐processing step, during which it is refined to produce more clinically favorable dose distributions (ppPD). Finally, the dose mimicking step employs a two‐phase optimization process, including fluence optimization and direct machine parameter optimization (DMPO), to reproduce the ppPD as closely as possible, tailored to the selected irradiation technique.

We are currently utilizing such an automated solution for VMAT‐based treatments of early‐stage breast cancer. Details regarding training data collection and curation, model training, and clinical implementation have been described elsewhere.^14^ For training, 80 manually designed VMAT plans were used, all employing a double‐reversed continuous partial arcs technique. This irradiation technique was also applied during the previous model validation (using 10 unseen patients) and in the creation of clinical auto‐plans, which, two years after validation, have been generated for over 100 patients. In this frame, the start and stop gantry angles were still manually selected by planners based on their clinical expertise to minimize irradiation of the patient's contra‐lateral side. Nonetheless, the irradiation technique used during dose mimicking does not necessarily have to match the one used to train the model. For example, reducing the arc span for VMAT plans or introducing fixed beams for IMRT plans may lead to deliverable dose distributions that differ from the predicted dose. In such cases, the mimicking algorithm optimizes arc or beam segments to approximate the ppPD as closely as possible, accounting for the physical and dosimetric limitations of the selected technique. This process may result in clinical doses with characteristics distinct from those predicted by a model trained exclusively with VMAT plans employing the same double‐reversed partial arc technique.

A practical strategy in post‐processing the PD is to enhance the overall plan quality by improving target coverage and homogeneity while minimizing OARs doses, thereby achieving the best possible dose distribution. This approach allows for exploring the potential of the mimicking algorithm to approximate such an ideal dose distribution using different irradiation techniques. For optimal results, gantry angles must also be optimized. An alternative approach to gantry angle optimization involves running dose mimicking for the ppPD with a full arc and then extracting the optimal directions by thresholding the fluence profile, resulting in a segment reduction technique that discards segments with low fluence.

In this paper, we present a novel automatic method for generating VMAT or IMRT plans for left‐sided breast cancer patients, featuring individualized arcs or beams optimized to reduce unintended OARs exposure. The proposed method relies on a monitor unit (MU)‐threshold‐based segment reduction strategy and can be seamlessy integrated into the existing clinical DL‐based auto‐planning workflow, enabling a fully automated process, including automated beam angle selection. We evaluated its impact on plan quality by comparing auto‐plans generated using this method to previously delivered clinical auto‐plans, in which the start and stop gantry angles of the double‐reversed partial arc technique were still manually selected by the user.

## 2 MATERIALS AND METHODS

### 2.1 Automated segment reduction

The automated segment reduction technique was integrated into the RayStation treatment planning system (TPS) v12a (RaySearch Laboratories, Stockholm, Sweden) as part of the DL‐based auto‐planning module.

**FIGURE 1:**
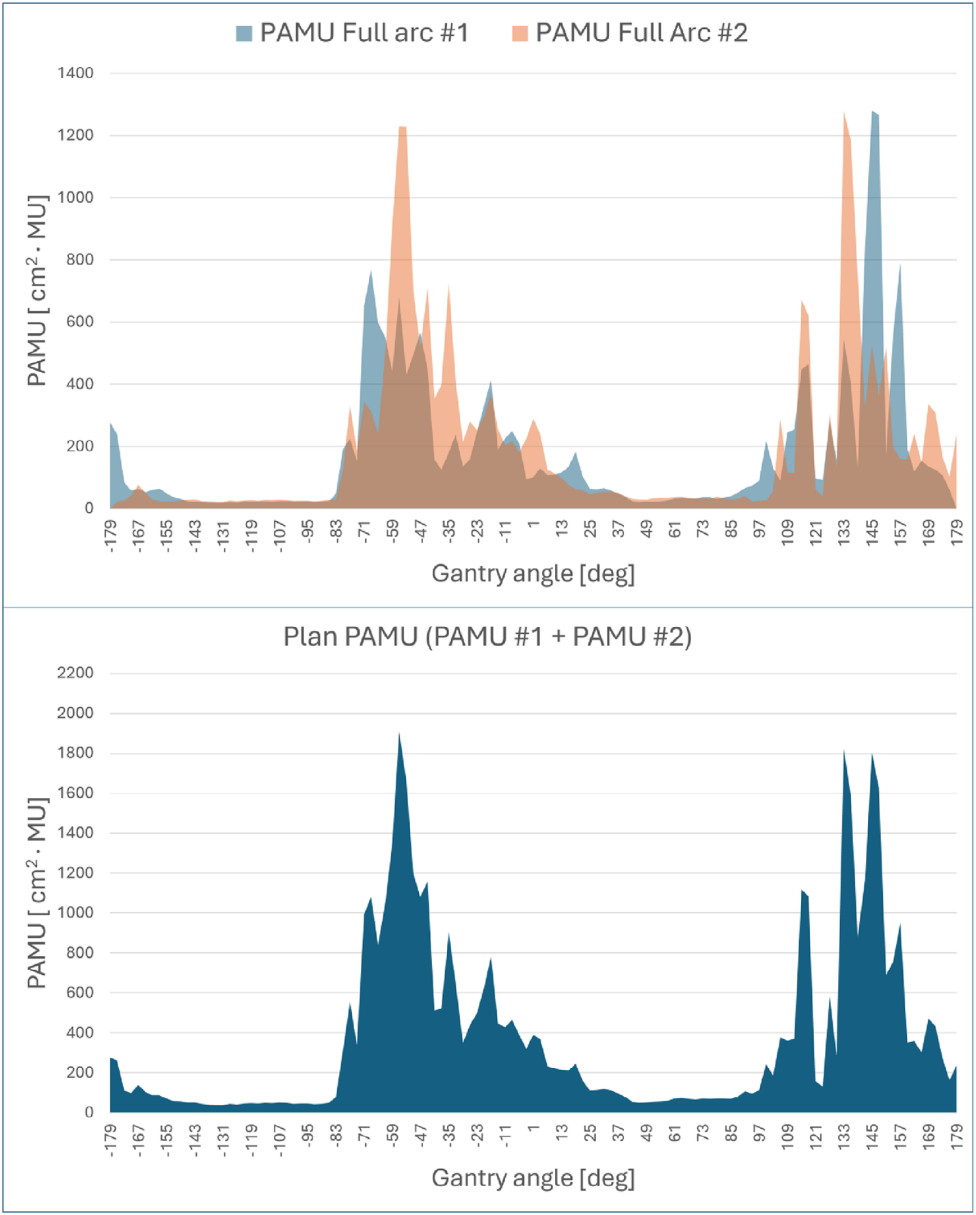
Upper panel: Product of segment Area and MU (PAMU, [cm^2^· MU]) values for each beam segment extracted from the mimicking of the post‐processed dose with two full reversed arcs. Lower panel: PAMU distribution obtained from the summation of the two full‐arc PAMU distributions, which was subsequently used for segment reduction via PAMU thresholding.

Two full dual arcs, with their isocenter positioned at the center of the breast PTV, were initially used to mimic the post‐processed predicted dose (ppPD) for each new patient. Upon completion of the mimicking process, monitor units (MU) and segment area (in cm^2^) were extracted from each control point (i.e., each segment), spaced three degrees apart. The product of irradiated area (cm^2^) and MU for each segment, referred to as PAMU (Product of segment Area and MU), was introduced as a quantitative metric for evaluating the dose delivered at each segment. Figure 1 shows the sum of PAMU values for each full arc for a given patient, which was subsequently used in the segment reduction method based on stepwise incremental thresholding criteria.

**FIGURE 2:**
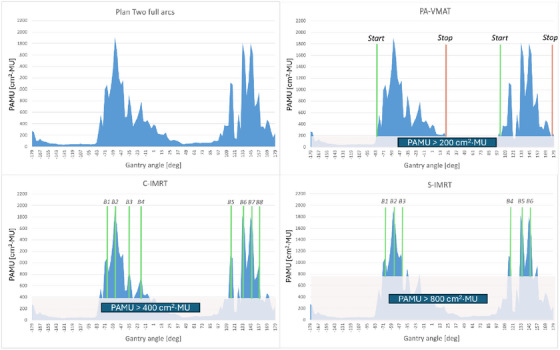
The three PAMU thresholding strategies for segment reduction applied to a typical patient. The start and stop angles are shown for PA‐VMAT (PAMU > 200 cm^2^·MU), an eight‐beam arrangement for C‐IMRT (PAMU > 400 cm^2^·MU), and a six‐beam arrangement for S‐IMRT (PAMU > 800 cm^2^·MU).

Three different PAMU thresholds were applied to create either partial arc VMAT or IMRT plans. Segments below these thresholds were eliminated, providing the start and stop angles for partial arc VMAT or the beam angles for the IMRT delivery. For partial arc VMAT (PA‐VMAT), a threshold of 200 cm^2^·MU was applied. A threshold of 400 cm^2^·MU was used for a complex IMRT (C‐IMRT) strategy. For this threshold, IMRT instead of VMAT was chosen, because of a minimum arc span limitation in the TPS. The beam angles were selected with a minimum spacing of 12 degrees between the beams. Finally, a threshold of 800 cm^2^·MU was used for a simple IMRT (S‐IMRT) with fewer beams compared to C‐IMRT (see Figure 2 for details). All three strategies were used to mimic the ppPD, resulting in deliverable plans.

### 2.2 Dose evaluation and plan comparison

Twenty left‐sided early breast cancer patients who had previously received radiation treatment under deep inspiration breath‐hold (DIBH) conditions were enrolled in the study. All treatment plans were designed using the DL‐based auto‐planning model in RayStation v12, which is routinely used in our clinic (described elsewhere^14^, ^15^). Briefly, the U‐Net model was trained on 80 VMAT plans, using a continuous dual partial arc delivery technique for simultaneous integrated boost (SIB) treatment of the PTV_Boost (lumpectomy region) and PTV_Breast, with doses of 48 Gy and 42.4 Gy in 16 fractions, respectively. The OARs were contoured using a commercially available DL‐based auto‐contouring model^23^ that relies on the ESTRO contouring guidelines,^24^ while both PTVs were created by adding 5 mm isotropic margins to the manually contoured clinical target volumes (CTVs), with a 3 mm retraction from the skin.

The continuous partial arc VMAT technique (CPA‐VMAT) was used as the reference, since it was the clinically delivered plan, and compared to PA‐VMAT, C‐IMRT, and S‐IMRT plans based on clinical goals listed in Table 1.

**TABLE 1:** List of clinical goals used for plan optimization and planning comparison. The percentage values for PTV boost and PTV breast are relative to their respective prescription doses, while for the PTV breast—PTV boost structure, they are relative to the prescription dose of PTV breast.

Structure	Figure of merit	Requirement
PTV_Boost	Mean dose	Between 47.5 Gy and 48.5 Gy
	D98%	≥ 45.6 Gy (95% of the prescribed dose)
	D2%	≤ 49.4 Gy (103% of the prescribed dose)
	Conformity index	≥ 0.6
PTV_Breast	D95%	≥ 40.3 Gy (95% of the prescribed dose)
	Conformity index	≥ 0.8
PTV_Breast—PTV_Boost	V44.5 Gy (105% of the prescribed dose)	≤ 10%
	V46.6 Gy (110% of the prescribed dose)	< 2%
Right Breast	Mean dose	≤ 2 Gy
	D1%	≤ 7 Gy
Heart	Mean dose	≤ 1.5 Gy
	D1%	≤ 6 Gy
Left Coronary Artery	D1%	≤ 6 Gy
Left Lung	Mean dose	≤ 6 Gy
	V5Gy	≤ 33%
	V20Gy	≤ 10%
Right Lung	Mean dose	≤ 1.5 Gy
	D1%	≤ 5 Gy
External—PTVs	V2Gy	≤ 18%
	V40.3 Gy	≤ 1%

CPA‐VMAT involved the use of two continuous patial arcs with the maximum allowed beam‐on time set to 75 s for each arc, for a total of 150 seconds per plan. For consistency, the same maximum beam‐on time was used for the PA‐VMAT plans, equally split between the two dual partial arcs (i.e., 37.5 s each arc). Both VMAT plans were optimized using three degrees spacing between control points. IMRT plans were optimized using a dynamic (sliding window) multi‐leaf collimator (MLC) segmentation technique. All plans were designed for the delivery on a C‐arm Synergy linac (Elekta AB, Stockholm, Sweden) equipped with a 160 MLC (80 leaves per bank) and a 5 mm width at the isocenter plane, and shared the same photon beam energy (6 MV FFF) and isocenter position. Finally, regardless of the mimicking technique, all plans were normalized such that the median PTV_Boost volume received the prescribed dose.

The plan ballistic resulting from the automated segment reduction techniques was compared between plans in terms of arc spans and start and stop gantry angles for VMAT, and in terms of beam numbers and gantry angle for IMRT. Finally, the total number of MU was evaluated across all plans.

The difference between the PD and the ppPD was evaluated to assess quality improvement during post‐processing. Additionally, the four different dose distributions were compared using the CPA‐VMAT as reference technique, and each one was also compared to the ppPD using the dose mimicking index (DMI) to determine which technique best approximated the ideal dose distribution through the mimicking algorithm:

(1) $$DMI=\sqrt{\sum_{i=1}^{N}\frac{1}{N}{\left(CG_{i}^{MD}-CG_{i}^{ppPD}\right)}^{2}},$$

where $CG_{i}^{MD}$ and $CG_{i}^{ppPD}$ represented the i‐th (N = 20, from Table 1) clinical goal median value (calculated for the whole cohort) resulting from the mimicked dose plan and the post‐processed predicted dose distribution, respectively. In the unlikely case of $CG_{i}^{MD}>CG_{i}^{ppPD}$ (for PTV coverage and conformity) and $CG_{i}^{\mathrm{MD}}<CG_{i}^{\mathrm{ppPD}}$(for PTV maximum dose and OARs dose), the difference between clinical goals was set to zero.

Statistical differences between treatment techniques were assessed using the non‐parametric Wilcoxon signed‐rank test (*p* < 0.05), as the results were not uniformly distributed, according to the Shapiro‐Wilk test.

## 3 RESULTS

### 3.1 Plan ballistic comparison

**FIGURE 3:**
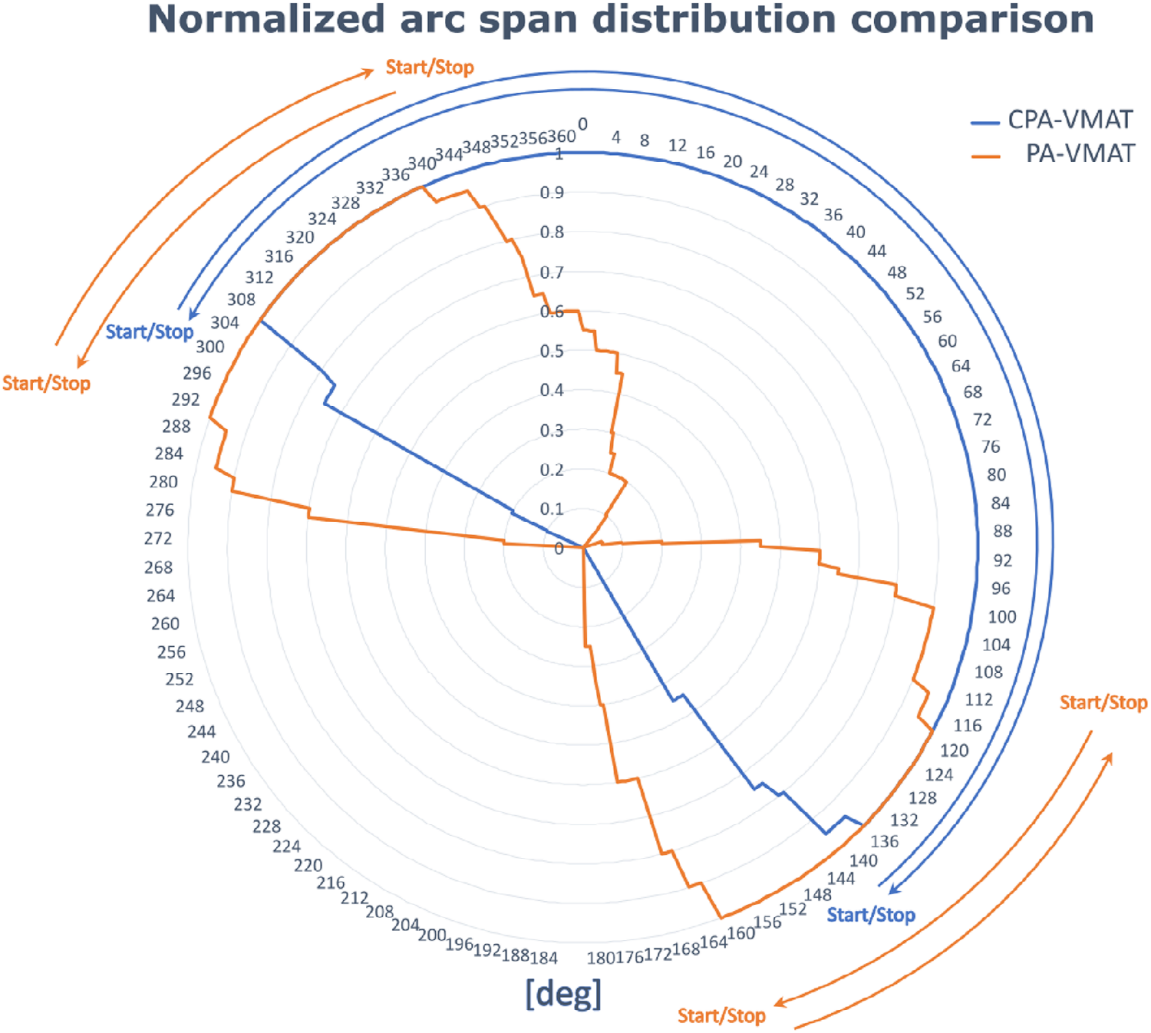
Comparison of start and stop beam angles between CPA‐VMAT (blue) and PA‐VMAT (orange). Beam angles were accumulated over 20 patients and normalized to unity. The plot highlights how the automated start angles for the internal and external partial arcs in PA‐VMAT differ significantly from the manually selected angles in CPA‐VMAT.

Optimal start/stop angles for PA‐VMAT had median values of 290°,340°, and 108°,170° for internal and external partial arcs, respectively, compared to 300°,150° for the continuous partial dual arc in CPA‐VMAT clinically used. The arc span distribution, accumulated and normalized over the 20 test patients, is reported in Figure 3. It shows the overall reduction in arc spans for PA‐VMAT compared to CPA‐VMAT, as well as the different initial and final positions of the gantry start and stop angles on both the internal and external treatment sides.

The gantry angle comparison is reported in Table 1 of the supplementary material for all 20 patients. For IMRT plans, the resulting median beam numbers were 8 (range 6–12) for C‐IMRT and 5 (range 4–9) for S‐IMRT. Beam angles, influenced by the PTV_Boost placement within the PTV_Breast, are detailed for every plan in Table 2 of the supplementary material. As expected, the largest number of monitor units (MUs) was required for IMRT plans. C‐IMRT plans had a median value of 1307 MUs for dose delivery, followed by S‐IMRT plans with 1182 MUs. The PA‐VMAT plans required approximately 50 fewer MUs than CPA‐VMAT plans (655 MUs vs. 710 MUs, median values). Both MU differences were statistically significant.

**TABLE 2:** Comparison of predicted dose (PD) and post‐processed predicted dose (ppPD) for each clinical goal, reported as median values and ranges across the 20 patients included in the study.

		Median value (range)		
Structure	Figure of merit	PD	ppPD	*p*‐value
PTV_Boost	Mean dose	47.8 Gy (47.8–47.9)	48 Gy (47.5–48.3)	< 0.001
	D98%	45.8 Gy (45.4–46.4)	46.3 Gy (45.3–46.5)	< 0.05
	D2%	48.7 Gy (48.4–49.0)	48.6 Gy (48–48.9)	0.44
	Conformity Index	0.69 (0.57–0.78)	0.87 (0.77–0.92)	< 0.001
PTV_Breast	D95%	40.6 Gy (40.1–41.1)	41.2 Gy (41–41.5)	< 0.001
	Conformity Index	0.92 (0.85–0.95)	0.89 (0.79–0.92)	< 0.001
PTV_Breast—PTV_Boost	V44.5 Gy	4.5 % (2.1–8.8)	2.2 % (1–4.7)	< 0.001
	V46.6 Gy	1 % (0.4–2.3)	0.6 % (0.2–1.5)	< 0.001
Right Breast	Mean Dose	0.8 Gy (0.5–1.3)	0.7 Gy (0.4–1.2)	< 0.001
	D1%	3.2 Gy (1.7–4.1)	3 Gy (1.7–4)	< 0.001
Heart	Mean Dose	0.9 Gy (0.6–1.1)	0.6 Gy (0.3–0.8)	< 0.001
	D1%	2.8 Gy (1.8–3.9)	2.2 Gy (1.5–3.3)	< 0.001
LAD	D1%	3.9 Gy (2.4–6.6)	2.3 Gy (1.7–2.3)	< 0.001
Left Lung	Mean Dose	4 Gy (2.9–4.7)	3.4 Gy (2.4–4)	< 0.001
	V5Gy	20.1 % (14.8–24.1)	16.1 % (12.2–19.6)	< 0.001
	V20Gy	5.5 % (2–7)	5.1 % (1.8–6.6)	< 0.001
Right Lung	Mean Dose	0.5 Gy (0.3–0.7)	0.4 Gy (0.2–0.6)	< 0.001
	D1%	2.2 Gy (1.2–3.2	2 Gy (1–3)	< 0.001
External—PTVs	V2Gy	11.8 % (9.5–17.5)	41.4 % (35.3–44.7)	< 0.001
	V40.3 Gy	0.2 % (0.2–0–3)	0.4 % (0.3–0.4)	< 0.001

### 3.2 Effect of post‐processing of the predicted dose

The differences between predicted dose (PD) and post‐processed predicted dose (ppPD) are reported in terms of median values and ranges in Table 2. It is noteworthy that the PD represents the output of a DL model trained with CPA‐VMAT plans, while the ppPD is the result of post‐processing (dose refinement) based on the original model settings and used as the reference dose for the mimicking algorithm.

As expected, the ppPD showed significant improvements over the PD in terms of median dose for most figures of merit for both PTVs and OARs, except for PTV_Boost D2%. Overall, target coverage and homogeneity improved. While dose‐volume differences were negligible for most OARs, clinically relevant reductions were observed for LAD D1% (−1.6 Gy) and left lung mean dose (−0.6 Gy, or −15%). Conversely, the ppPD performed worse than PD in tissues outside the PTVs and OARs (i.e., the External‐PTVs ROI). This discrepancy was due to the intentional addition of a uniform low‐dose background of approximately 17 Gy during post‐processing, ensuring the mimicking algorithm did not penalize doses from valid angles, even if no predicted dose was present in that region. This was an ad hoc solution introduced to facilitate optimization. Figure S1 in the supplementary material compares the dose distributions between PD and ppPD for a representative patient.

### 3.3 Plan comparison

Table 3 compares dose distributions from the four different mimicking techniques, with a boxplot comparison provided in Figure S2 and S3 of the supplementary material. In the following, all presented differences are statistically significant unless otherwise stated.

**TABLE 3:** Comparison of planned doses across four alternative planning techniques used to replicate the post‐processed predicted dose. Asterisks (*) denote statistically significant differences (*p* < 0.05) between the candidate techniques and the CPA‐VMAT technique.

		Median value (range)			
Structure	Figure of merit	CPA—VMAT	PA‐ VMAT	C—IMRT	S—IMRT
PTV_Boost	Mean dose	47.9 Gy (47.8–48)	47.9 Gy (47.8–48.1)	47.9 Gy (47.8–48)	47.9 Gy (47.8–48.1)
	D98%	46.1 Gy (45.7–46.4)	46.2 Gy (45.5–46.6)	46.3 Gy (46.1–46.6) *	46.4 Gy (46.1–46.9) *
	D2%	48.9 Gy (48.7–49.3)	49 Gy (48.7–49.4)	48.8 Gy (48.6–49.2)	49.1 Gy (48.6–49.8)
	CI	0.66 (0.48–0.72)	0.62 (0.52–0.72)	0.63 (0.56–0.76)	0.57 (0.36–0.75) *
PTV_Breast	D95%	40.7 Gy (40–41.2)	41 Gy (40.6–41.3) *	41.1 Gy (40.8–41.3) *	41.1 Gy (40.7–41.6) *
	CI	0.90 (0.82–0.93)	0.90 (0.80–0.92) *	0.88 (0.80–0.93) *	0.87 (0.69–0.92) *
PTV_Breast—PTV_Boost	V44.5 Gy	7.7 % (3.9–10.6)	7.1 % (4.7–16.2)	6.3 % (2.4–11.9)	9.1 % (2.7–40.8) *
	V46.6 Gy	1.4 % (0.7–2.4)	1.3 % (0.8–3.1)	1.2 % (0.6–3.3)	1.9 % (0.7–5.3) *
Right Breast	Mean dose	0.8 Gy (0.5–1.5)	0.6 Gy (0.3–1.1) *	0.9 Gy (0.5–1.6)	0.7 Gy (0.4–1.5) *
	D1%	3.1 Gy (1.7–6.4)	3.2 Gy (1.9–4.9)	3.3 Gy (2.2–5.4) *	3.4 Gy (2–6.7)
Heart	Mean dose	1.1 Gy (0.8–1.7)	0.8 Gy (0.4–1.2) *	1 Gy (0.5–1.7) *	0.8 Gy (0.4–1.7) *
	D1%	3.6 Gy (2.7–5.2)	2.8 Gy (1.7–4.6) *	3 Gy (2–5) *	2.9 Gy (1.7–5.2) *
LAD	D1%	4.7 Gy (3.5–6.1)	4.2 Gy (2.6–5.4) *	4.3 Gy (2.8–5.6) *	4.6 Gy (2.6–6)
Left Lung	Mean dose	4.6 Gy (3.3–5.9)	4.1 Gy (2.8–5.2) *	4 Gy (2.8–5.6) *	4 Gy (2.9–5.5) *
	V5Gy	21.3 % (16.3–27.6)	19.4 % (13.8–25.1) *	19.9 % (14.9–27.7) *	19.9 % (13.5–27.3) *
	V20Gy	6.8 % (2.7–9.1)	6 % (2.5–7.5) *	5.5 % (2.5–9) *	6.5 % (3–9.7)
Right lung	Mean dose	0.6 Gy (0.4–0.9)	0.3 Gy (0.1–0.4) *	0.4 Gy (0.2–0.8) *	0.3 Gy (0.2–0.9) *
	D1%	2.2 Gy (1.6–3.7)	1.2 Gy (0.8–1.8) *	1.8 Gy (1.3–2.2) *	1.6 Gy (1–2.6) *
External—PTVs	V2Gy	16 % (12.8–22.8)	13.9 % (9.7–20) *	13.2 % (9.2–21.7) *	12.3 % (6.8–18.8) *
	V40.3 Gy	0.3 % (0.2–0.5)	0.3 % (0.2–0.5) *	0.4 % (0.3–0.6) *	0.4 % (0.3–0.7) *

Concerning the targets, both IMRT techniques provided better results in terms of PTV_Boost coverage at the cost of losing in PTV_Boost conformity for S‐IMRT. Similarily, for PTV_Breast, all the alternative techniques provided better coverage, with the only PA‐VMAT that did not show a worst conformity that was instead observed for both IMRT techniques. As expected, due to the limited number of beams involved, the S‐IMRT resulted the worst technique in terms of dose homogeneity with an increased value of hotspots within the breast volume excluding the SIB.

Excluding a few exceptions, all the alternative irradiation techniques demonstrated to be superior to the CPA‐VMAT for OARs sparing. In detail, PA‐VMAT was always superior to CPA‐VMAT except for the right breast D1% for which results were similar, reducing the OARs maximum dose especially for the heart, LAD, and right lung as well as the mean dose for the left lung. Similar results were achieved by the C‐IMRT technique although the planned dose for the right breast and the heart was slightly larger than PA‐VMAT. The S‐IMRT showed an overall good OARs sparing as well, even though the maximum dose to the LAD and the left lung V20Gy resulted in similar values compared to the CPA‐VMAT ones.

**FIGURE 4:**
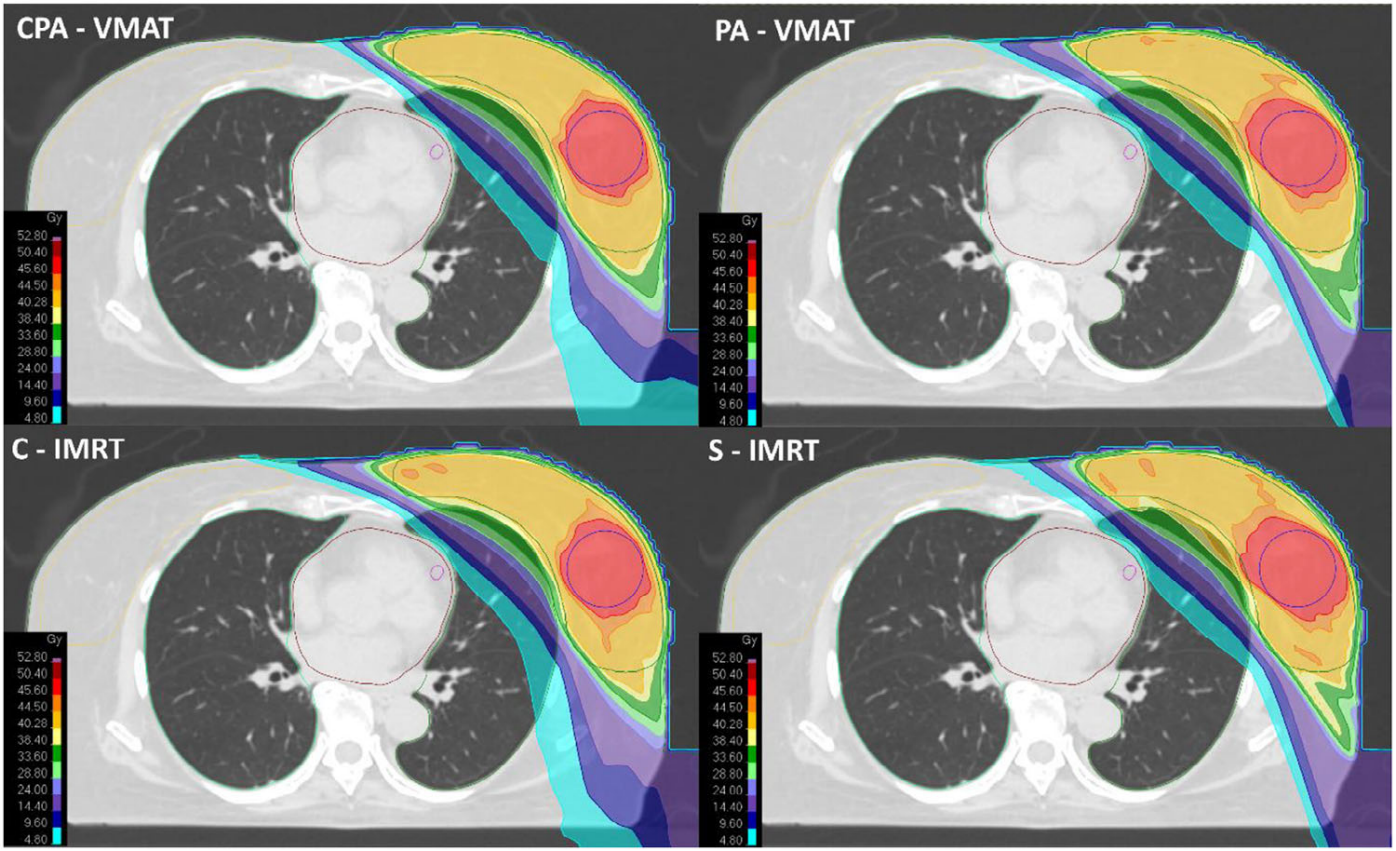
Comparison of the axial dose distribution across the four alternative techniques for a typical patient.

Figure 4 shows a dose distribution comparison in the axial plane for a typical case, while the DVH comparison averaged over the 20 test patients is reported in Figure S4 of the supplementary material.

Out of 400 evaluated clinical goals, the number of failed goals was 18, 8, 14, and 37 for CPA‐VMAT, PA‐VMAT, C‐IMRT, and S‐IMRT, respectively. The mean number of failed clinical goals per plan was 0.9, 0.5, 0.7, and 1.9. Most failures were observed for PTV_Boost dose conformity (*n* = 28), breast volume hotspots (*n* = 23), and low‐dose normal tissue exposure (*n* = 8). The full list of failed occurrences and corresponding dose‐volume deviations is in Table S3 of the supplementary material.

### 3.4 DMI results

The DMI results are shown in Table 4. Overall, the PA‐VMAT technique exhibited the smallest DMI value, suggesting the best mimicking approximation of the ppPD, while the reference CPA‐VMAT had the highest DMI, indicating the worst approximation. To further analyze the mimicking approximation, we computed DMI values separately for PTVs (DMI PTVs) and OARs (DMI OARs). In this case, C‐IMRT demonstrated the best approximation for PTVs, whereas PA‐VMAT achieved the best approximation for OARs. CPA‐VMAT was the worst technique for both ppPD approximations.

**TABLE 4:** Dose mimicking index (DMI) comparison listed in terms of global, PTVs only, and OARs only for the four alternative irradiation techniques. Lowest values of DMI correspond to best approximation of the post‐processed predicted dose after dose mimicking.

Technique	DMI global	DMI PTVs	DMI OARs
CPA—VMAT	65	20.9	81.7
PA—VMAT	46.7	15.5	58.5
C—IMRT	48.4	7.8	61.7
S—IMRT	53.7	16.4	67.5

The median value of $CG_{i}^{\mathrm{MD}}$ resulted superior in terms of plan quality than the $CG_{i}^{\mathrm{ppPD}}$ in 8 out of the 80 median clinical goals evaluated (20 clinical goals, 4 techniques). This occurred for the average dose to the right breast in PA‐VMAT and S‐IMRT techniques, and for both the average and maximum doses to the right lung in PA‐VMAT, C‐IMRT, and S‐IMRT. These results were primarily attributed to the artificial increase in ppPD on the contralateral side, as mentioned in the previous section. This adjustment had a greater impact on corresponding clinical goals for contralateral OARs.

## 4 DISCUSSION

We presented in this paper an automated segment reduction technique aimed at improving the quality of dose distribution for DL‐based automated treatment planning of early‐stage breast cancer. This technique was seamlessly integrated into a commercially available auto‐planning pipeline, providing alternative irradiation techniques beyond those used for model training. It enabled optimal arc span reduction for VMAT treatments and facilitated the transition to IMRT‐based treatments, leveraging irradiation techniques different from those used during model training.

Regardless of the irradiation geometry employed, the proposed technique provided appropriate gantry angles, including start/stop arc beam angles or beam directions. These angles were automatically selected based on patient geometry and the position of the SIB within the breast volume. The selection of optimized gantry angles relied on a thresholding method that exploited intensity profiles derived from mimicking the post‐processed predicted dose (ppPD), initially computed using a full‐arc VMAT geometry.

The rationale for this approach stems from the mimicking algorithm itself, which is used as the optimization algorithm in the final step of the automated planning workflow. This algorithm ensures that the deliverable dose is the best possible approximation of the ppPD according to the selected irradiation technique, even if the technique is suboptimal (e.g., compared to two full arcs). In the version of the RayStation TPS (v12) used, the mimicking algorithm employed a set of structure‐based functions to penalize dose voxels exceeding the ppPD.^25^ These objectives were applied to all structures involved in the optimization, ensuring dose control across all voxels within the CT scan. These objectives were built after model training and became an integral part of the auto‐planning package being model‐based. This level of approximation could not be guaranteed by a “classic” inverse planning optimization algorithm that attempts to minimizes a cost function that typically consist of a sum of dose‐volume objectives. For instance, using the same two arcs optimization technique, missing or suboptimal objectives defined for the contralateral side could result in higher doses to contralateral structures, undermining the effectiveness of the proposed method.

**FIGURE 5:**
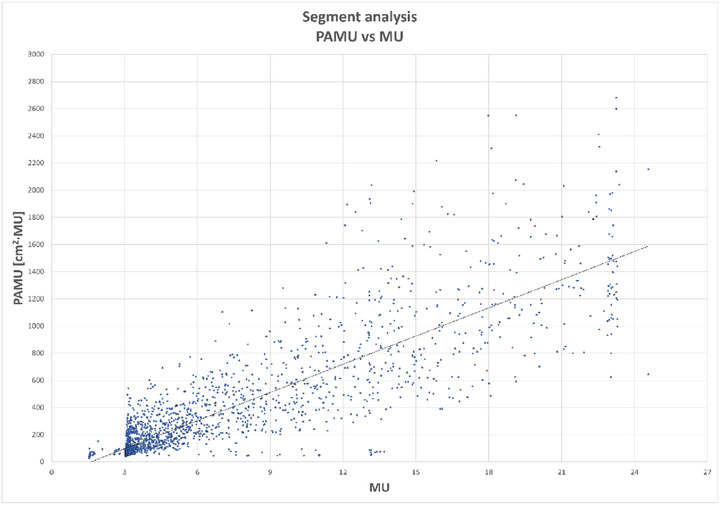
Product of segment Are and MU (PAMU) plotted against the MU for all the segments extracted from the dual full arc beam arrangement for the 20 patients involved in the study. The solid line represents the linear fit of the plotted data.

We chose the product of segment area and MU (PAMU) as the thresholding metric for segment reduction, as it maximized the delivery information contained in each segment. Segments with large areas, even when associated with a few MU, could still significantly contribute to the overall planned dose distribution. We initially evaluated two thresholding strategies: MU‐only and PAMU. Analyzing the segments from 20 full‐arc plans, we observed a supra‐linear relationship between PAMU and MU (Figure 5). Thresholding based solely on MU would have excluded numerous segments with low MU but large areas, leading to suboptimal beam angle extraction and reduced effectiveness of the method.

The PAMU thresholds were determined by analyzing the PAMU profiles extracted from full‐arc mimicking. These profiles exhibited a characteristic pattern influenced by the prescribed dose (defining the PAMU range) and the SIB position within the irradiated volume. Notably, the PAMU profiles featured two prominent peaks corresponding to tangential irradiation from both internal and external breast sides, with valleys in between. An initial PAMU threshold of 200 cm^2^·MU was chosen to eliminate segments in these valleys that contributed minimally to the total dose.

The linac beam model used in this study does not support turning off the dose rate during VMAT delivery. Consequently, a low dose rate was maintained, and the MLCs were kept as closed as possible for segments not actively contributing to dose delivery, leading to a form of PAMU “noise.” Segments corresponding to this noise were effectively removed using the 200 cm^2^·MU threshold.

As a next step, we increased the PAMU threshold by a factor of two to extract a profile that selected segments contributing most significantly to the total dose. However, the TPS's minimum arc span requirement of 24 degrees sometimes limited further arc span reduction. To address this, we used an equi‐spaced sampling technique with at least 12 degrees between IMRT beams. This technique was applied to the second threshold level and the highest threshold level to generate IMRT plans when arc span reduction was constrained.

Interestingly, the dose mimicking of the post‐processed predicted dose (ppPD) for PA‐VMAT and C‐IMRT outperformed the results obtained with CPA‐VMAT, the irradiation technique learned by the model and currently used in our clinical practice. This demonstrates that the irradiation geometry influences the quality of dose mimicking, highlighting the importance of using a refined dose for mimicking purposes. The geometry impacts dose mimicking due to the mechanical and dosimetric limitations of the delivery system. As mentioned previously, continuous partial arcs still deliver a few MUs even when not required, due to linac limitations, increasing low‐dose spillage for all OARs. Conversely, the limited number of beams in S‐IMRT may be suboptimal for mimicking the ppPD, sometimes causing large hotspots within target volumes. Notably, three of the ten cases where hot spot tolerance was violated for the S‐IMRT technique used 3–5 beams, suggesting that too few beams were employed to ensure optimal mimicking. Without post‐processing, the after‐mimicking planned dose would likely have been inferior, as the mimicking process would have tried to replicate the predicted dose instead.

The use of an optimization algorithm—whether based on inverse planning with a proper formulation of objectives or mimicking—employed as a second stage in the auto‐planning process to generate fluence maps or segment apertures, provides a more versatile solution compared to DL models that are trained with both dose distribution and treatment geometry (i.e., DICOM RT Plan). As demonstrated in this study, different treatment strategies can yield clinical dose distributions superior to those used during model training. Otherwise, for new patients, the DL model will typically use the learned RT plan information, producing a dose distribution less flexible to beam geometry variation unless the model is updated and retrained with plans using different geometric irradiation ballistics.

Focusing on the plan comparison, the PA‐VMAT technique seems to be the best choice among the four evaluated, considering the trade‐off between plan quality and beam‐on time, while also returning the lowest global DMI. In terms of PTV coverage, uniformity, and conformity, C‐IMRT was superior to all other techniques (see DMI target results), but it required the highest number of MUs for plan delivery. However, C‐IMRT could still be an option in clinics where VMAT is not available or when limited by TPS licensing.

Several studies have compared breast irradiation techniques without nodal involvement, and while fractionation regimens and the use of SIB may differ, comparisons with our study are relevant. Zhang et al.^10^ compared hybrid IMRT (h‐IMRT) to CPA‐VMAT for breast treatments under free breathing, finding that CPA‐VMAT resulted in higher mean doses to the heart, contralateral breast, and both lungs compared to h‐IMRT. This trend aligns with the comparison between CPA‐VMAT and our IMRT plans, though their differences were larger, likely due to the absence of DIBH. In a multicenter study^5^ involving DIBH but no SIB, the mean doses to the heart, ipsilateral lung, and contralateral breast were 1.6, 6.6, and 0.9 Gy, respectively, for a partial arc technique similar to ours, using the same treatment machine. Our PA‐VMAT plans yielded improved results for the same OARs (0.8, 4.1, and 0.6 Gy), suggesting how much plan quality may be improved using auto‐planning, even without post‐processing. The superiority of small partial arcs over large ones has been demonstrated in previous studies using RapidArc, where beam angles to avoid OARs irradiation were manually selected.^7^ The ratios between mean doses for OARs such as the heart, ipsilateral lung, and contralateral breast in those studies ranged from 0.5 to 0.88, which aligns with our PA/CPA‐VMAT ratios of 0.75, 0.7, and 0.89, respectively.

The main limitation of this study lies in the PAMU thresholding criteria, which were defined based on PAMU profiles and characteristics specific to the treatment scenario (such as dose fractionation, machine limitations, and clinical indications). This could limit the general applicability of the criteria to other treatment sites or fractionation regimens. However, the fundamental principle of evaluating and discriminating intensity profiles to improve dose delivery efficiency by removing unnecessary segments can still be applied to different clinical scenarios by adjusting the thresholding criteria.

Additionally, the proposed method was effective when the mimicking algorithm was used as the optimization algorithm. However, it still requires validation with a more formal inverse planning problem formulation.

Another limitation is that the results are restricted to the patient anatomy used for model training, particularly the breast volume, which may be strongly correlated with OAR doses. Although discussing the necessary data curation strategy to improve DL model performance^26^, ^27^ is beyond the scope of this paper, it is important to note that the method depends on the initial output of the DL model—the predicted dose. Therefore, applying the method outside the training distribution (e.g., to patients with larger breast volumes than those in the training dataset) would likely lead to sub‐optimal dose predictions, potentially altering the expected results.

## 5 CONCLUSION

This study introduces an automated segment reduction technique that enhances radiotherapy planning for early‐stage breast cancer by optimizing dose distribution and reducing arc spans in VMAT treatments. Integrated into a commercial auto‐planning pipeline, the technique ensures optimal gantry angles and minimizes unnecessary beam segments, improving treatment efficiency.

The PA‐VMAT technique outperformed CPA‐VMAT, offering better OAR sparing, lower beam‐on time, and the best mimicking dose approximation. While C‐IMRT provided superior target coverage, it required more MUs. Smaller arcs and optimized segment reduction improve plan quality.

## AUTHOR CONTRIBUTION

**Michele Zeverino**: Conceptualization; data curation; formal analysis; investigation; methodology; resources; software; writing—original draft; writing—review & editing. **Gian Guyer**: Software; validation; writing—review & editing. **Wendy Jeanneret‐Sozzi**: Validation; writing—review & editing. **Fernanda Herrera**: Validation; writing—review & editing. **François Bochud**: Conceptualization; validation; writing—review & editing. **Raphaël Moeckli**: Conceptualization; methodology; project administration; supervision; validation; writing—original draft; writing—review & editing.

## CONFLICT OF INTEREST STATEMENT

The authors declare no conflicts of interest.

## Supporting information



Suppporting Information
