# Supplementary material for: An automated patient‐specific segment reduction‐based beam angle optimization technique for deep learning auto‐planning for early breast cancer
Source: J Appl Clin Med Phys. 2025 Oct 14;26(10):e70293. doi: 10.1002/acm2.70293 (PMC12521044; doi:10.1002/acm2.70293)
Supplement: Supplementary file 1 — Suppporting Information [file ACM2-26-e70293-s001.doc]

| Patient # | CPA-VMAT | | PA-VMAT | | | |
| --- | --- | --- | --- | --- | --- | --- |
|  | 2 reversed continuos full arcs | | Internal reversed 2 partial arcs | | External reversed 2 partial arcs | |
|  | Start [deg] | Stop [deg] | Start [deg] | Stop [deg] | Start [deg] | Stop [deg] |
| 1 | 300 | 140 | 290 | 10 | 97 | 174 |
| 2 | 305 | 145 | 280 | 0 | 85 | 166 |
| 3 | 297 | 150 | 280 | 352 | 91 | 160 |
| 4 | 300 | 140 | 274 | 13 | 88 | 163 |
| 5 | 300 | 135 | 277 | 15 | 88 | 172 |
| 6 | 297 | 145 | 280 | 337 | 95 | 166 |
| 7 | 295 | 145 | 277 | 348 | 88 | 172 |
| 8 | 305 | 145 | 280 | 345 | 113 | 166 |
| 9 | 300 | 150 | 277 | 346 | 90 | 179 |
| 10 | 300 | 150 | 274 | 34 | 100 | 179 |
| 11 | 300 | 150 | 277 | 349 | 100 | 160 |
| 12 | 300 | 140 | 277 | 13 | 82 | 163 |
| 13 | 305 | 145 | 277 | 349 | 91 | 166 |
| 14 | 305 | 150 | 274 | 37 | 73 | 179 |
| 15 | 300 | 150 | 274 | 13 | 88 | 179 |
| 16 | 295 | 142 | 283 | 34 | 85 | 175 |
| 17 | 300 | 150 | 277 | 343 | 88 | 172 |
| 18 | 305 | 140 | 277 | 4 | 118 | 172 |
| 19 | 300 | 150 | 277 | 40 | 97 | 179 |
| 20 | 300 | 150 | 277 | 19 | 97 | 175 |

Table 1S. Start and stop gantry angle comparison between the two VMAT techniques. For the CPA-VMAT the same start and stop angles were used for the two reversed continuous partial arcs. For the PA-VMAT, the same start and stop angles were used for the two partial arcs on each side, internal and external.

| Patient # | Technique | Beam #1  [deg] | Beam #2  [deg] | Beam #3  [deg] | Beam #4  [deg] | Beam #5  [deg] | Beam #6  [deg] | Beam #7  [deg] | Beam #8  [deg] | Beam #9  [deg] | Beam #10  [deg] | Beam #11  [deg] | Beam #12  [deg] |
| --- | --- | --- | --- | --- | --- | --- | --- | --- | --- | --- | --- | --- | --- |
| 1 | C-IMRT | 292 | 309 | 325 | 100 | 117 | 134 | 150 | - | - | - | - | - |
|  | S-IMRT | 295 | 305 | 317 | 121 | 133 | - | - | - | - | - | - | - |
| 2 | C-IMRT | 283 | 297 | 312 | 325 | 100 | 115 | 131 | 146 | 163 | - | - | - |
|  | S-IMRT | 283 | 297 | 310 | 103 | 134 | 152 | - | - | - | - | - | - |
| 3 | C-IMRT | 280 | 295 | 310 | 100 | 115 | 130 | 145 | 160 | - | - | - | - |
|  | S-IMRT | 289 | 301 | 112 | 127 | 136 | - | - | - | - | - | - | - |
| 4 | C-IMRT | 280 | 295 | 310 | 325 | 106 | 121 | 136 | 151 | 163 | - | - | - |
|  | S-IMRT | 289 | 310 | 300 | 118 | 155 | - | - | - | - | - | - | - |
| 5 | C-IMRT | 280 | 297 | 314 | 118 | 133 | 148 | 163 | - | - | - | - | - |
|  | S-IMRT | 292 | 310 | 124 | 139 | 301 | - | - | - | - | - | - | - |
| 6 | C-IMRT | 283 | 296 | 310 | 109 | 127 | 143 | 160 | - | - | - | - | - |
|  | S-IMRT | 304 | 289 | 127 | 136 | - | - | - | - | - | - | - | - |
| 7 | C-IMRT | 280 | 297 | 315 | 94 | 109 | 142 | 157 | 172 | - | - | - | - |
|  | S-IMRT | 122 | 286 | 310 | 127 | 139 | 154 | - | - | - | - | - | - |
| 8 | C-IMRT | 289 | 306 | 322 | 121 | 133 | 148 | - | - | - | - | - | - |
|  | S-IMRT | 301 | 316 | 142 | - | - | - | - | - | - | - | - | - |
| 9 | C-IMRT | 283 | 298 | 313 | 127 | 145 | 163 | - | - | - | - | - | - |
|  | S-IMRT | 292 | 305 | 317 | 145 | - | - | - | - | - | - | - | - |
| 10 | C-IMRT | 280 | 297 | 314 | 331 | 113 | 130 | 144 | 158 | 172 | - | - | - |
|  | S-IMRT | 289 | 319 | 112 | 133 | 145 | 163 | 175 | - | - | - | - | - |
| 11 | C-IMRT | 280 | 300 | 319 | 106 | 127 | 145 | - | - | - | - | - | - |
|  | S-IMRT | 289 | 304 | 112 | 127 | 142 | - | - | - | - | - | - | - |
| 12 | C-IMRT | 283 | 296 | 310 | 91 | 105 | 119 | 133 | 147 | 160 | - | - | - |
|  | S-IMRT | 289 | 307 | 100 | 112 | 124 | 136 | 148 | 160 | - | - | - | - |
| 13 | C-IMRT | 288 | 306 | 94 | 109 | 127 | 144 | - | - | - | - | - | - |
|  | S-IMRT | 289 | 307 | 118 | 144 | - | - | - | - | - | - | - | - |
| 14 | C-IMRT | 286 | 303 | 320 | 337 | 24 | 83 | 97 | 115 | 139 | 155 | 169 | - |
|  | S-IMRT | 289 | 305 | 318 | 330 | 82 | 108 | 144 | 166 | - | - | - | - |
| 15 | C-IMRT | 277 | 292 | 307 | 322 | 337 | 127 | 142 | 157 | - | - | - | - |
|  | S-IMRT | 280 | 292 | 310 | 325 | 109 | 139 | - | - | - | - | - | - |
| 16 | C-IMRT | 279 | 294 | 309 | 324 | 339 | 97 | 112 | 127 | 142 | 157 | - | - |
|  | S-IMRT | 289 | 301 | 313 | 103 | 118 | 130 | 145 | 160 | - | - | - | - |
| 17 | C-IMRT | 283 | 297 | 313 | 328 | 106 | 116 | 129 | 142 | 157 | - | - | - |
|  | S-IMRT | 289 | 308 | 328 | 109 | 121 | 136 | 151 | - | - | - | - | - |
| 18 | C-IMRT | 283 | 295 | 307 | 319 | 331 | 132 | 154 | 169 | - | - | - | - |
|  | S-IMRT | 292 | 310 | 325 | 132 | 157 | - | - | - | - | - | - | - |
| 19 | C-IMRT | 289 | 303 | 318 | 332 | 346 | 358 | 100 | 115 | 130 | 145 | 160 | 175 |
|  | S-IMRT | 295 | 307 | 316 | 352 | 118 | 127 | 145 | 166 | 175 | - | - | - |
| 20 | C-IMRT | 289 | 304 | 319 | 335 | 352 | 115 | 129 | 143 | 157 | - | - | - |
|  | S-IMRT | 289 | 307 | 325 | 116 | 133 | 143 | 157 | - | - | - | - | - |

Table 2S. Gantry angles for IMRT techniques. starting with the innermost (the most internal one) and proceeding outward to the outermost (the most external one).


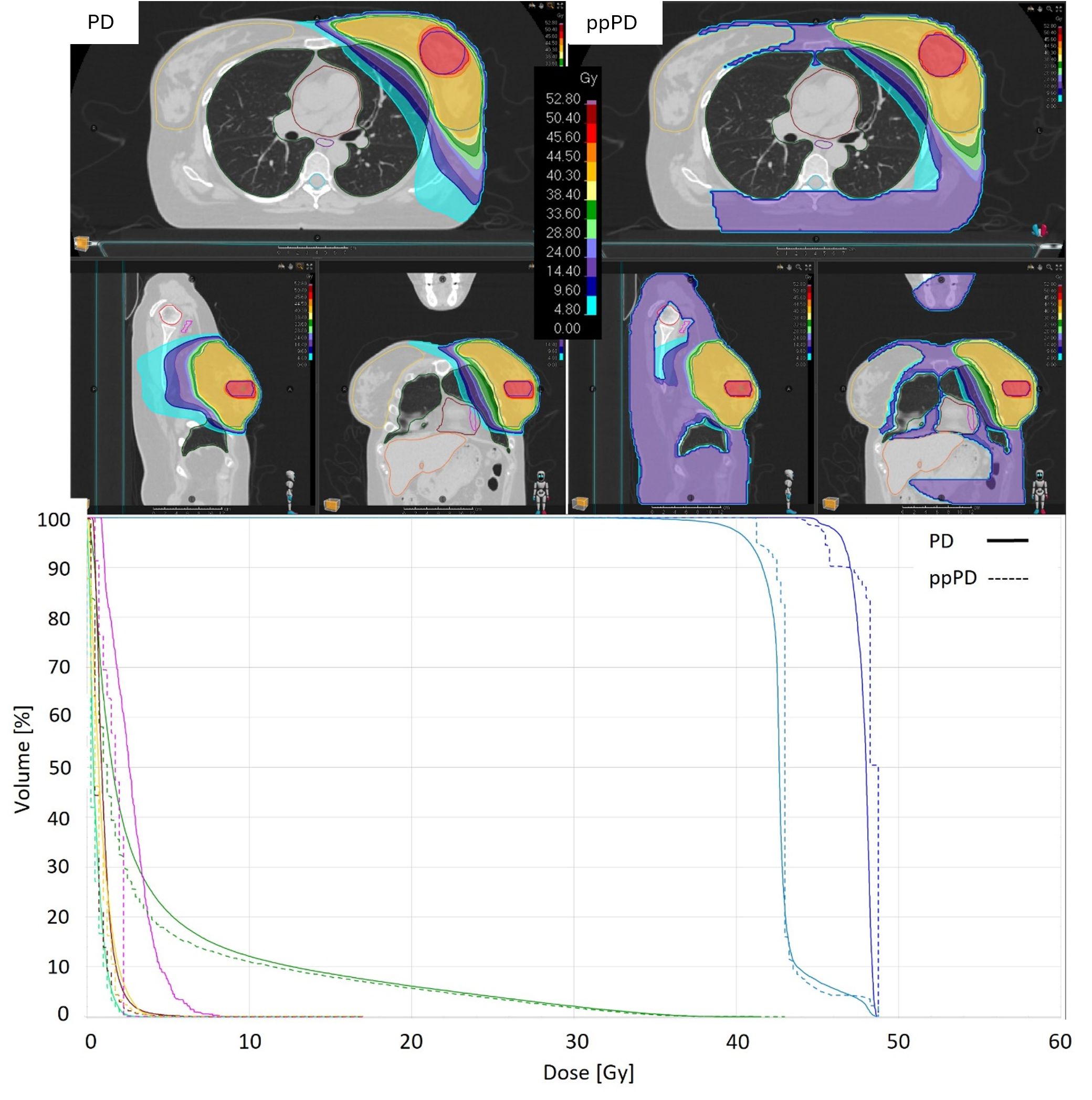


Figure1S. Dose distribution and DVH comparison between predicted dose (PD) and post-processed PD (ppPD) for one example case. DVH shows curves for PTV_Boost (dark blue), PTV_Breast (light blue), left lung (dark green), right lung (light green), heart (brown), right breast (yellow), and left coronary (pink).


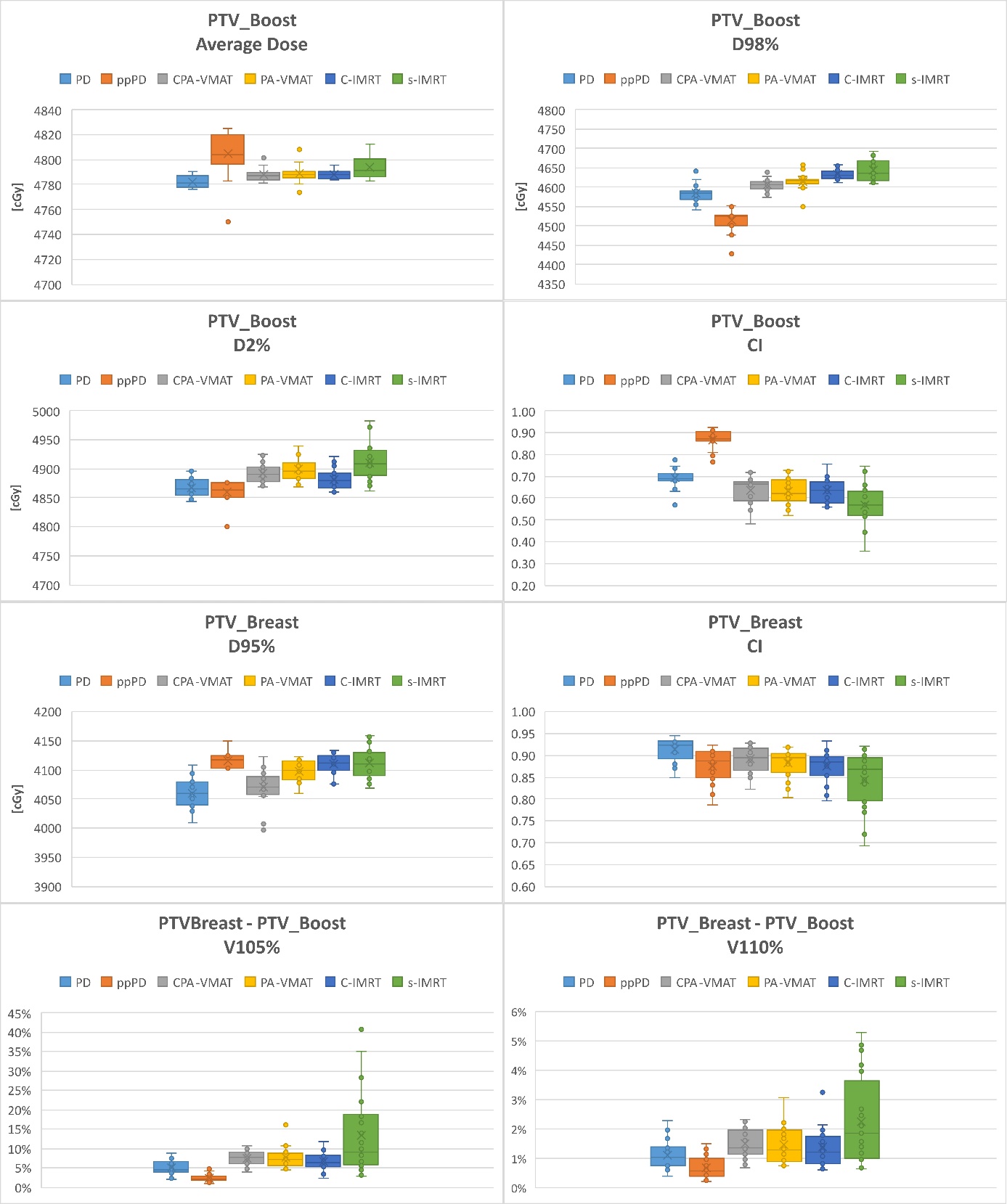


Figure 2S. Planned dose comparison showing the predicted dose (PD), the post-processed PD (ppPD), and the mimicked dose arising from the four alternative beam geometries for both the PTVs and the PTV_Breast – PTV_Boost structure.


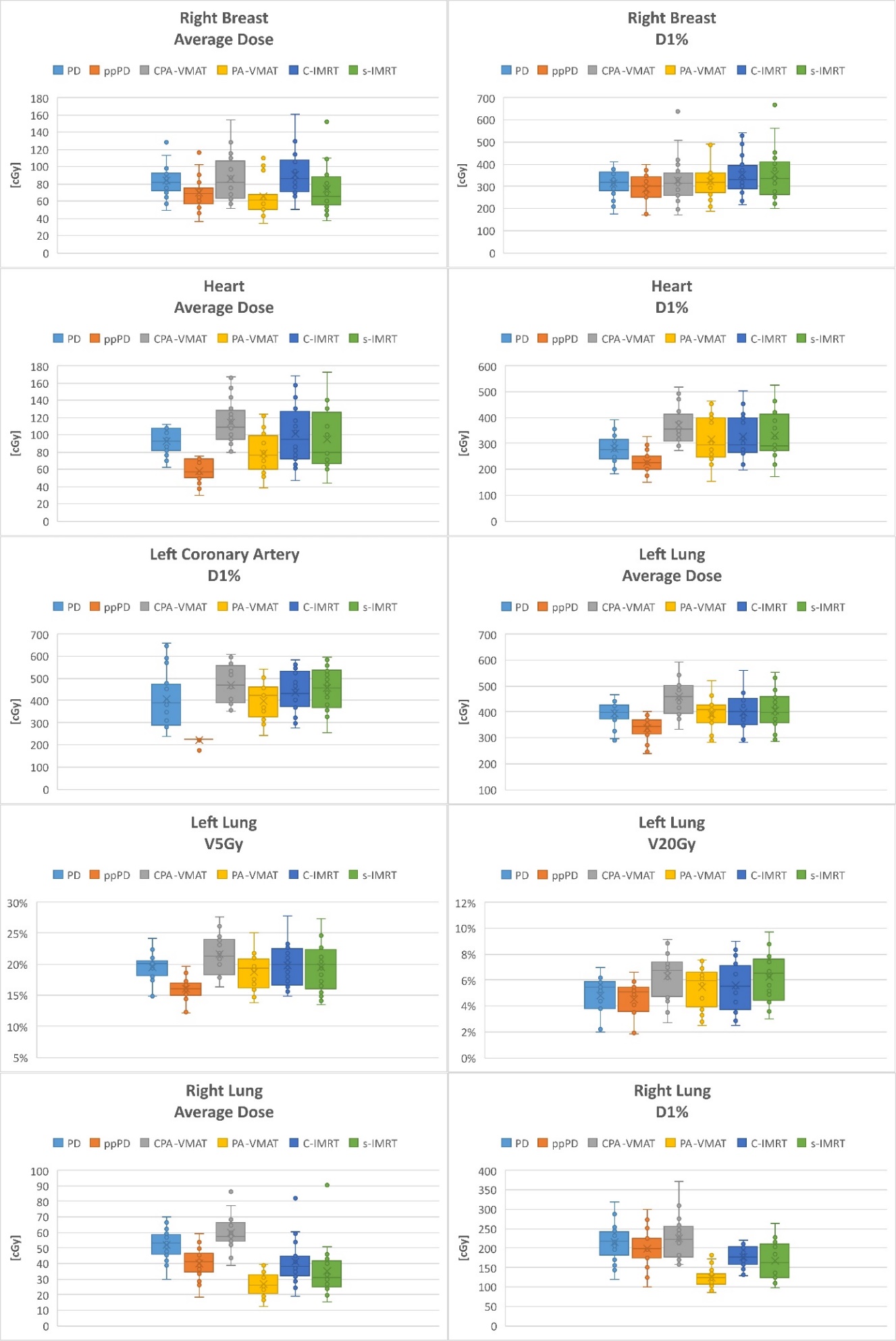


Figure 3S. Planned dose comparison showing the predicted dose (PD), the post-processed PD (ppPD), and the mimicked dose arising from the four alternative beam geometries for all OARs involved in the study.


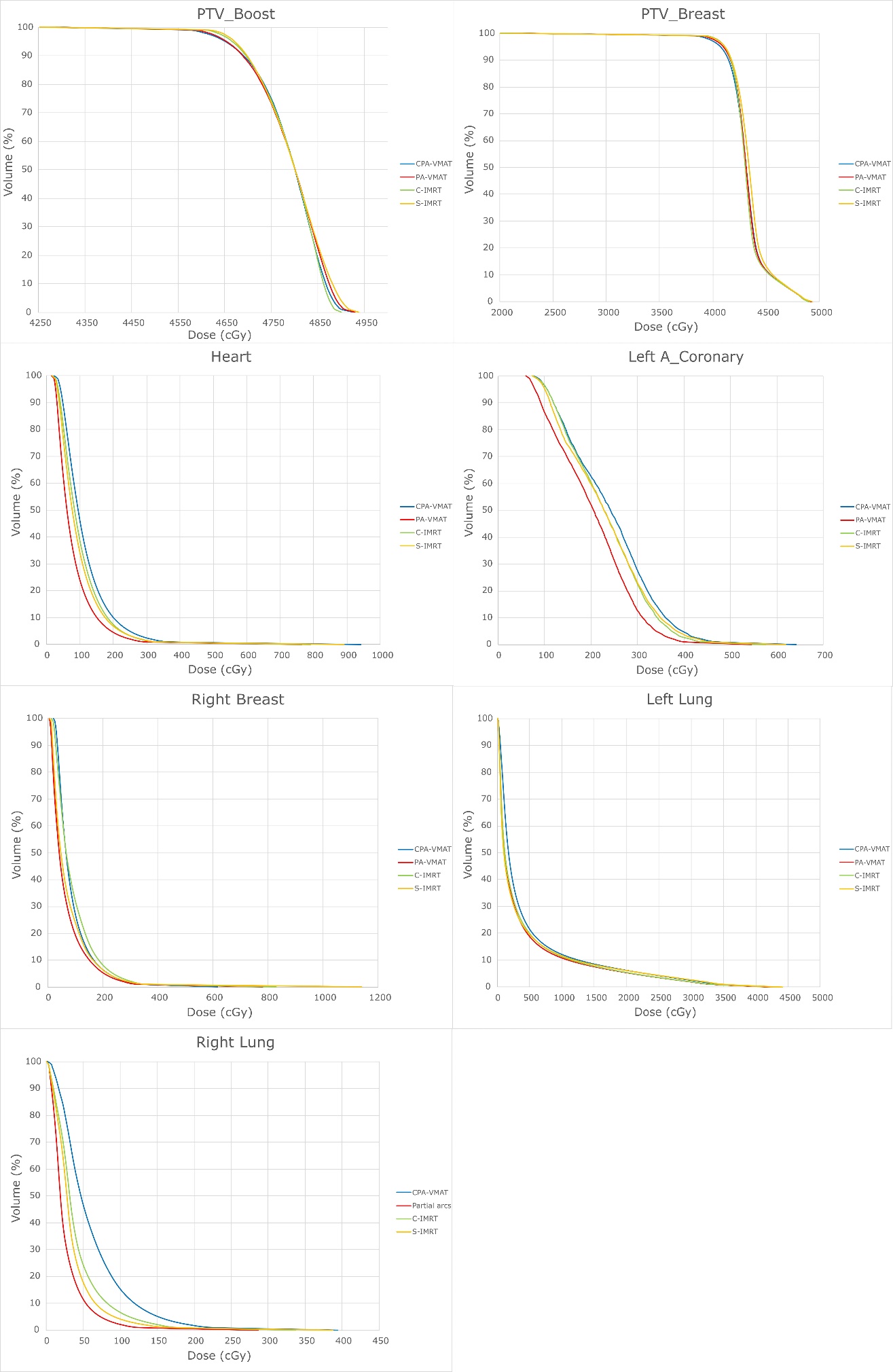


Figure 4S. Mean DVH comparison averaged over the 20 test patients for each irradiation technique: CPA-VMAT (blue), PA-VMAT (red), C-IMRT (green), and S-IMRT (yellow).

| Structure | Figure of merit | Requirement | Failed Occurrences  (mean deviation from requirement) | | | |
| --- | --- | --- | --- | --- | --- | --- |
|  |  |  | CPA-VMAT | PA-VMAT | C-IMRT | S-IMRT |
| PTV_Boost | Mean Dose | 48 Gy ± 0.5 Gy | 0 | 0 | 0 | 0 |
|  | D98% | ≥ 45.6 Gy | 0 | 1 (-0.1 Gy) | 0 | 0 |
|  | D2% | ≤ 49.4 Gy | 0 | 0 | 0 | 2 (+0.3 Gy) |
|  | Conformity Index | ≥ 0.6 | 5 (-0.05) | 4 (-0.05) | 6 (-0.07) | 13 (-0.08) |
| PTV_Breast | D95% | ≥ 40.3 Gy | 2 (-0.3 Gy) | 0 | 0 | 0 |
|  | Conformity Index | ≥ 0.8 | 0 | 0 | 0 | 5 (-0.03) |
| PTV_Breast – PTV_Boost | V44.5 Gy | ≤ 10% | 0 | 1 (+0.6 %) | 1 (+0.2 %) | 8 (+0.14 %) |
|  | V46.6 Gy | < 2% | 2 (+0.3 %) | 1 (+1.1 %) | 2 (+0.8%) | 8 (+1.8 %) |
| Right Breast | Mean Dose | ≤ 2 Gy | 0 | 0 | 0 | 0 |
|  | D1% | ≤ 7 Gy | 0 | 0 | 0 | 0 |
| Heart | Mean Dose | ≤ 1.5 Gy | 3 (+0.1 Gy) | 0 | 3 (+0.1 Gy) | 1 (+0.2 Gy) |
|  | D1% | ≤ 6 Gy | 0 | 0 | 0 | 0 |
| Left Coronary Artery | D1% | ≤ 6 Gy | 1 (+ 0.1 Gy) | 0 | 0 | 0 |
| Left Lung | Mean Dose | ≤ 6 Gy | 0 | 0 | 0 | 0 |
|  | V5Gy | ≤ 33% | 0 | 0 | 0 | 0 |
|  | V20Gy | ≤ 10% | 0 | 0 | 0 | 0 |
| Right Lung | Mean Dose | ≤ 1.5 Gy | 0 | 0 | 0 | 0 |
|  | D1% | ≤ 5 Gy | 0 | 0 | 0 | 0 |
| External - PTVs | V2Gy | ≤ 18% | 5 (+3.2 %) | 2 (+1.5 %) | 1 (+3.7 %) | 0 |
|  | V40.3 Gy | ≤ 1% | 0 | 0 | 0 | 0 |

Table 3S. Comparison of the number of failed occurrences and mean dose deviation (when applicable) calculated with respect to the clinical goal requirement.
